# 

**Published:** 1845-12-01

**Authors:** 


AUSTIN FLINT, M. D.

**Vol. 1.** **BUFFALO, DECEMBER, 1845.** **No. 7.**

### CONTENTS:

### ORIGINAL COMMUNICATIONS.

Notes of an European Tour. By F. H. HAMILTON, M. D., Prof. of Surgery in Geneva Medical College. P. 145
Rupture of an Ovarian Cyst by a fall; escape of its contents into peritoneal cavity; absorption, and radical cure. By JAMES P. WHITE, M. D. - 151
Case of Omental Hernia; removal of the Sac, with spermatic cord, and testicle. By ALDEN S. SPRAGUE, M. D. - 153
Case of Typhoid Fever, with Autopsy and remarks by G. N. BURWELL, M. D. - 156
Letter from Accoucheur. No. 3. - 160

### EDITORIAL, MEDICAL INTELLIGENCE, ETC.

Physicians in Buffalo. - 162

Treatment of Aneurism by compression. - 163

Electro Physiology. - 164

Case of Placenta Praevia, treated by Prof. Simpson's method. - 166

Our Exchange Journals. - 166

Medical Remembrancer, or Book of Emergencies. - 167

Monograph on Strabismus, by F. H. Hamilton, M. D - 167

Treatise on Hare lip, by Dr. S. P. Hullihen. - 167

Injury of head, &c., operation of trephining, by D. Brainard, M. D. - 168

Medical Fees, No. 3. - 168

Medical Legislation. - 168

BUFFALO:
C. F. S. THOMAS, PRINTER AND PUBLISHER,
Exchange Buildings, Third Story, Main Street.

